# Supplementary material for: Targeting USP2 regulation of VPRBP-mediated degradation of p53 and PD-L1 for cancer therapy
Source: Nat Commun. 2023 Apr 6;14:1941. doi: 10.1038/s41467-023-37617-3 (PMC10079682; doi:10.1038/s41467-023-37617-3)
Supplement: Supplementary file 1 — Supplementary information file [file 41467_2023_37617_MOESM1_ESM.pdf]

Supplemental Information

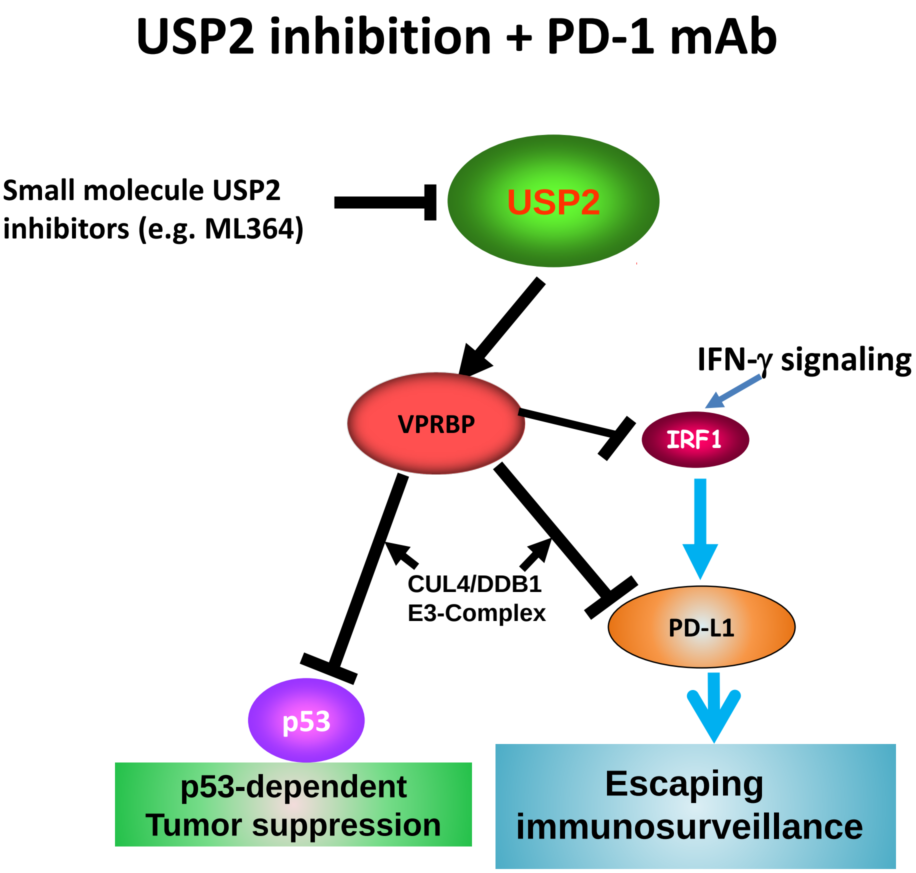

Supplementary Fig. 1. A working model for USP2 inhibition in regulating p53 activity and the PD1/PD-L1 immune checkpoint (see text).

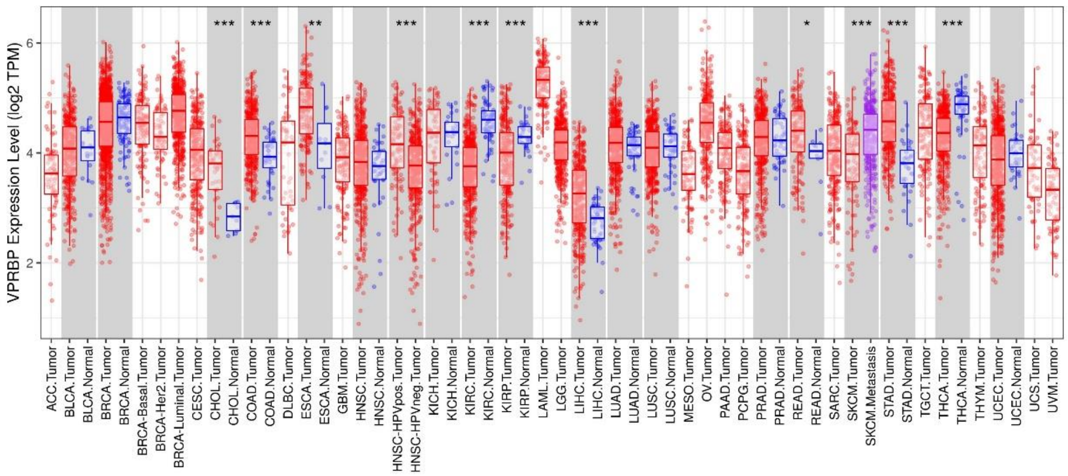

Supplementary Fig. 2. VPRBP TCGA mRNA expression of paired tumor and normal tissues. VPRBP is significantly overexpressed in a subset of cancers: Chol, Coad, ESCA, LIHC, STAD, SKCM met vs tumor. Red: Tumor; Blue: Normal tissue. n=10897 tissues, Wilcoxon test, * p<0.05; ** p<0.01; *** p<0.001. Source data and exact *p value* are provided in the Source data file.

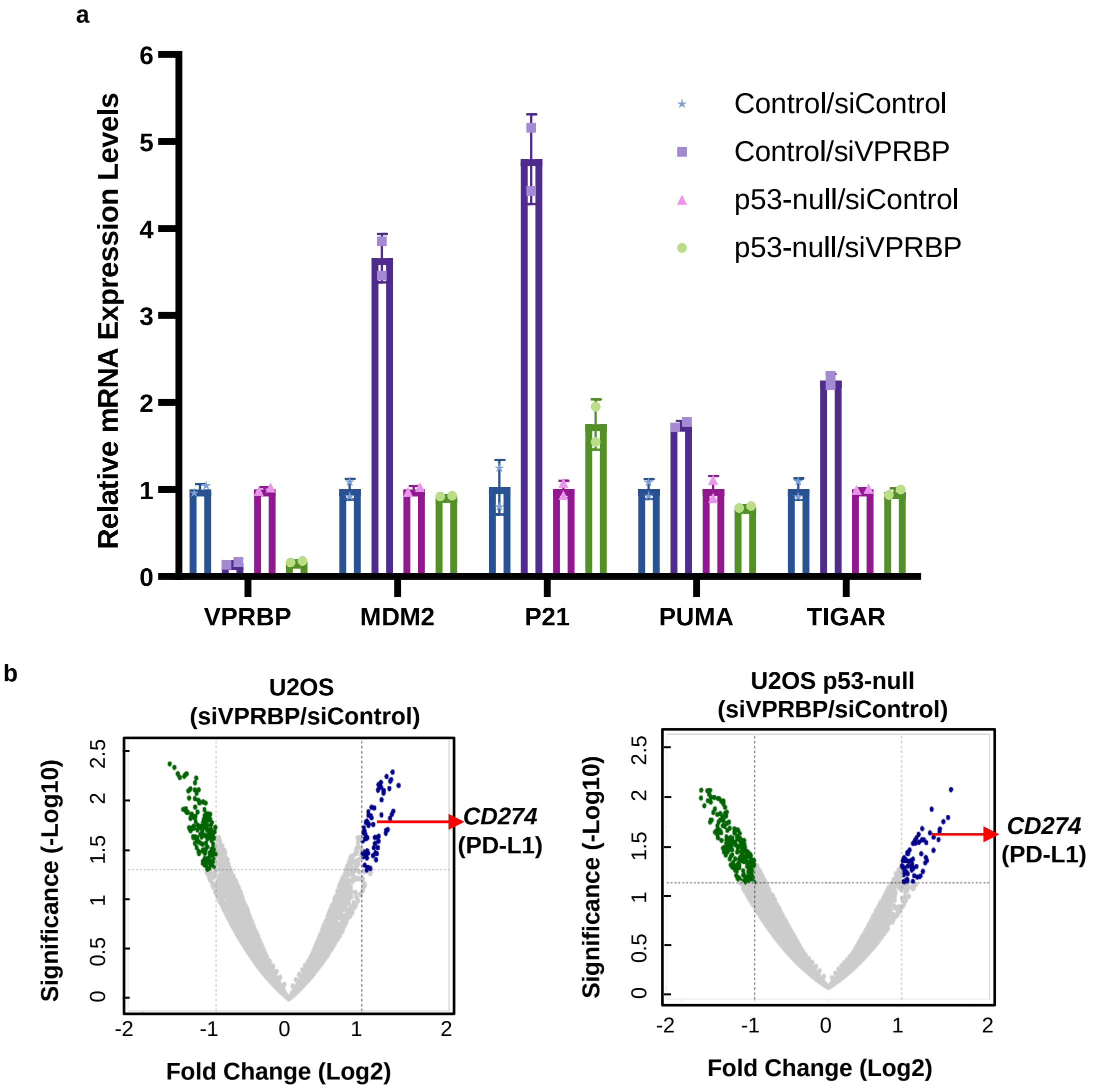

Supplementary Fig. 3. a, Representative qRT-PCR analysis of U2OS crispr control or p53-null cells that were transfected with the control or VPRBP siRNA. n=2 biologically independent samples, mean±SD. b, Volcano plot of differentially expressed genes in U2OS and U2OS p53-null cells. Green dot: down-regulated gene; Blue dot: up-regulated gene; Gray dot: no-change gene. Source data are provided in the Source data file.

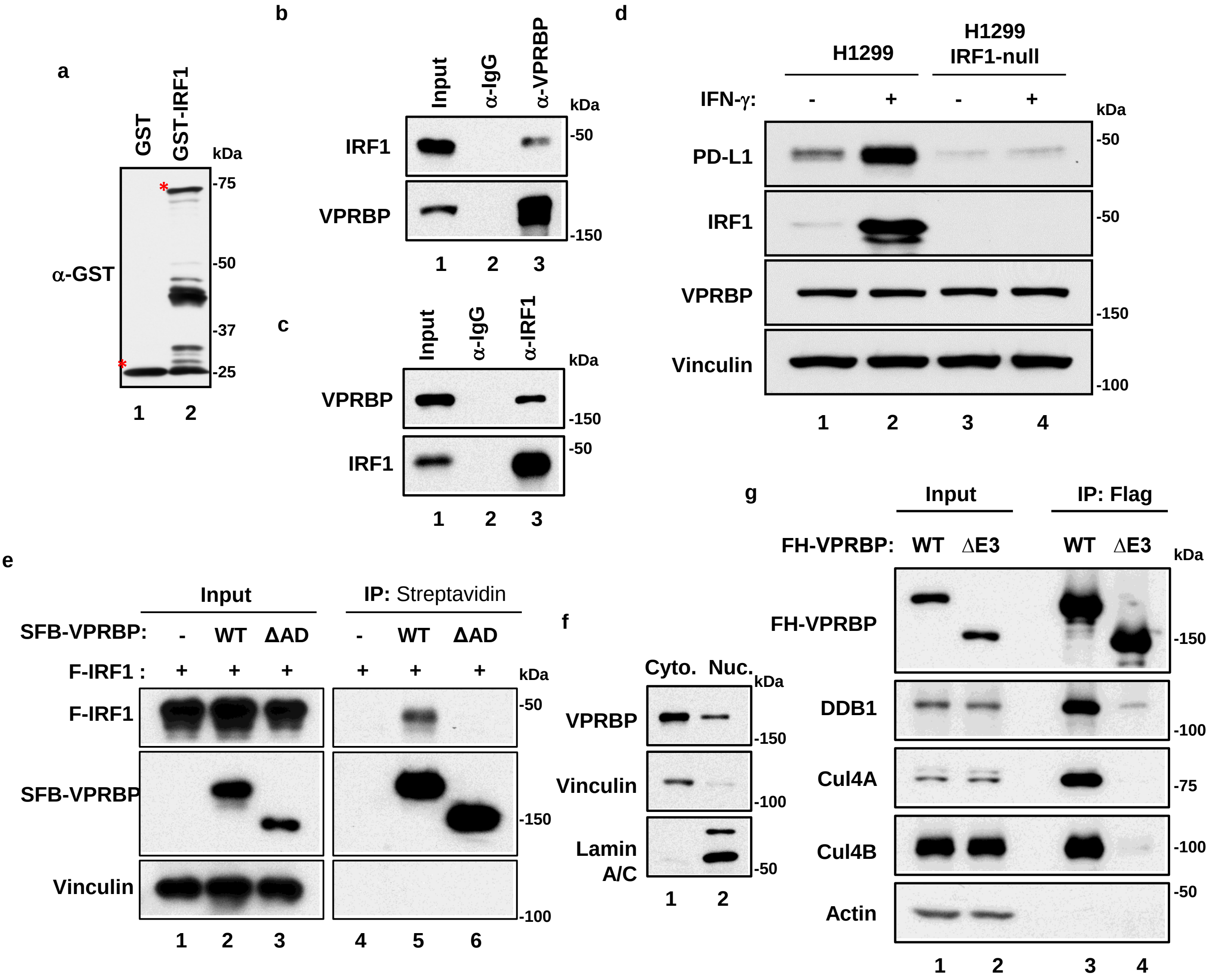

Supplementary Fig. 4. a, Purified GST and GST-IRF1 protein for GST-pulldown assay. "*" indicates specific band. b, Western blot analysis for endogenous IRF1 after immunoprecipitation of endogenous VPRBP in A549 cells. c, Western blot analysis for endogenous VPRBP after immunoprecipitation of endogenous IRF1 in HEK293 cells. d, Western blot analysis of relative PD-L1 levels in H1299 parental and *IRF1*-null cells were treated by 10ng/ml of Interferon-gamma (IFN-g) for 24h. e, HEK293 cells were transfected with F-IRF1 alone, or plus SFB-VPRBP wildtype or ΔAD constructs. After IP with streptavidin beads, input and immunoprecipitates were analyzed by western blot. f, Western blot analysis of cytosolic and nuclear extracts in HEK293T cells. g, Western blot analysis of DDB1, Cul4A and Cul4B in input and anti-Flag immunoprecipitates in HEK293 cells transfected with FH-VPRBP wildtype and ΔE3 constructs. Data are representative of two independent experiments. Source data are provided in the Source data file.

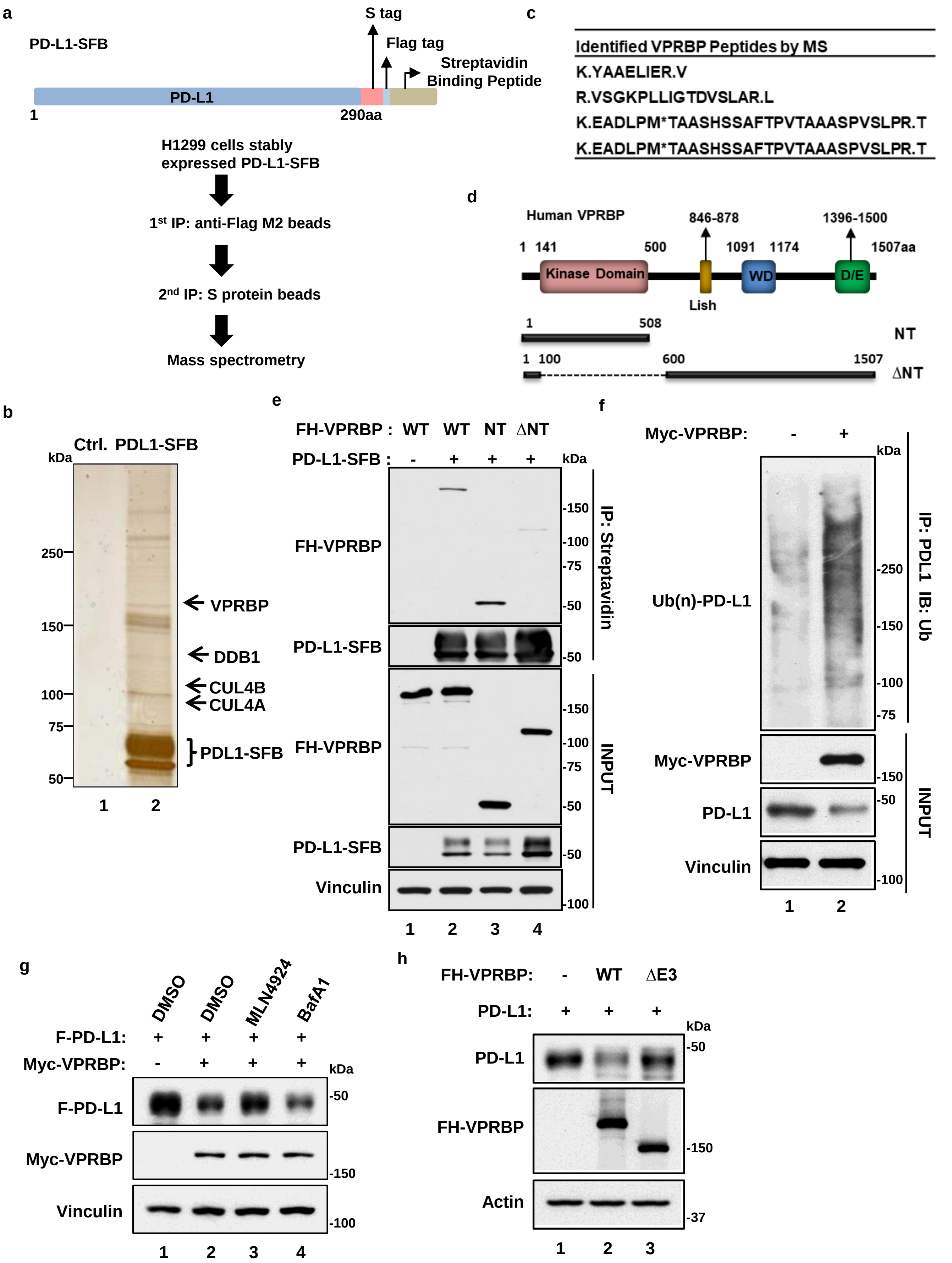
a
PD-L1-SFB
S tag
Flag tag
Streptavidin Binding Peptide
PD-L1
1
290aa
H1299 cells stably expressed PD-L1-SFB
1st IP: anti-Flag M2 beads
2nd IP: S protein beads
Mass spectrometry
c
Identified VPRBP Peptides by MS
K.YAAELIER.V
R.VSGKPLLIGTDVSLAR.L
K.EADLPM*TAASHSSAFTPVTAAASPVSLPR.T
K.EADLPM*TAASHSSAFTPVTAAASPVSLPR.T
d
Human VPRBP
846-878
1396-1500
1 141
500
1091 1174
1507aa
Kinase Domain
WD
D/E
Lish
1
508
NT
1 100
600
1507
ΔNT
b
Ctrl. PDL1-SFB
kDa
250
150
100
75
50
VPRBP
DDB1
CUL4B
CUL4A
PDL1-SFB
1 2
e
FH-VPRBP : WT WT NT ΔNT
PD-L1-SFB : - + + +
kDa
FH-VPRBP
PD-L1-SFB
FH-VPRBP
PD-L1-SFB
Vinculin
-150
-100
-75
-50
-50
-150
-100
-75
-50
-50
-100
IP: Streptavidin
INPUT
1 2 3 4
f
Myc-VPRBP: - +
kDa
Ub(n)-PD-L1
-250
-150
-100
-75
IP: PDL1 IB: Ub
Myc-VPRBP
PD-L1
Vinculin
-150
-50
-100
INPUT
1 2
g
DMSO DMSO MLN4924 BafA1
F-PD-L1: + + + +
Myc-VPRBP: - + + +
kDa
F-PD-L1
Myc-VPRBP
Vinculin
-50
-150
-100
1 2 3 4
h
FH-VPRBP: - WT ΔE3
PD-L1: + + +
kDa
PD-L1
FH-VPRBP
Actin
-50
-150
-37
1 2 3

Supplementary Fig. 5. a, Schematic illustration of PD-L1-SFB protein and the tandem affinity purification-mass spectrometry. b, Silver staining of control and PDL1-SFB complexes which were obtained by tandem affinity purification using streptavidin sepharose and S-protein agarose. c, VPRBP peptides identified in PD-L1-SFB complex by mass spectrometry. d, Schematic illustration of human VPRBP full-length protein and deletion mutants. e, Western blot analysis of PD-L1-SFB immunoprecipitates in H1299 cells transfected with indicated Flag-HA-VPRBP deletions. f, Determine the poly-ubiquitination of endogenous PD-L1 in H1299 cells transfected with indicated constructs for 24h and treated with 5uM MG132 for additional 12h. g, Western blot analysis of HEK293 cells transfected with constructs as indicated followed by DMSO, 1μM MLN4924 and 1μM BafA1 treatment for additional 24h. h, Western blot analysis for F-PD-L1 in HEK293 cells that were transfected with F-PD-L1 alone, or F-PD-L1 plus FH-VPRBP wildtype (WT) or ΔE3 for 24h. Data are representative of two independent experiments. Source data are provided in the Source data file.

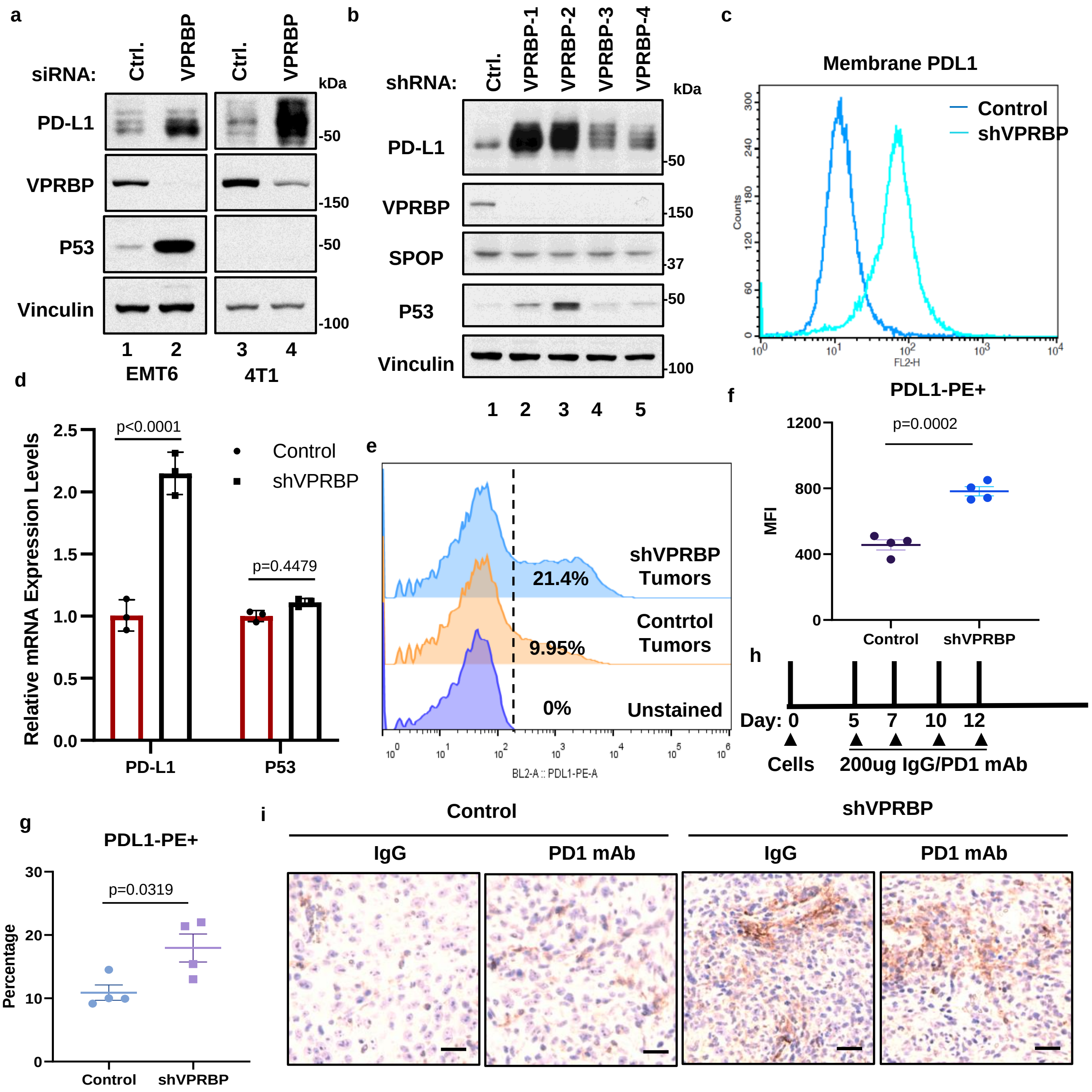

Supplementary Fig. **6.** a, Western analysis of whole cell extracts of EMT6 and 4T1 cells transfected with control or VPRBP siRNA for 72h. b, Western analysis of EMT6 cells transduced with or without different VPRBP shRNA lentiviruses. a and b, Data are representative of two independent experiments. c, FACS analysis of membrane PD-L1 in EMT6 control and shVPRBP cells. n=2 images per cohort. d, QRT-PCR analysis of PD-L1 and P53 mRNA levels in EMT6 control and shVPRBP cells. n=3 biologically independent samples, mean±SD, two-way ANOVA. e, Representative FACS data of PD-L1 in EMT6 control and shVPRBP tumors. n=4 images per cohort. f and g, Mean fluorescence intensity (MFI) and the percentage of PD-L1+ cells in EMT6 control and shVPRBP tumors. n=4 tumors, mean±SEM, two-way unpaired t test. h, Treatment plan in EMT6-implanted Balb/c mice for i. i, Representative immunohistochemistry images of PDL1 staining in EMT6 tumors. Scale bar:25μM. n=3 images per group. Source data are provided in the Source data file.

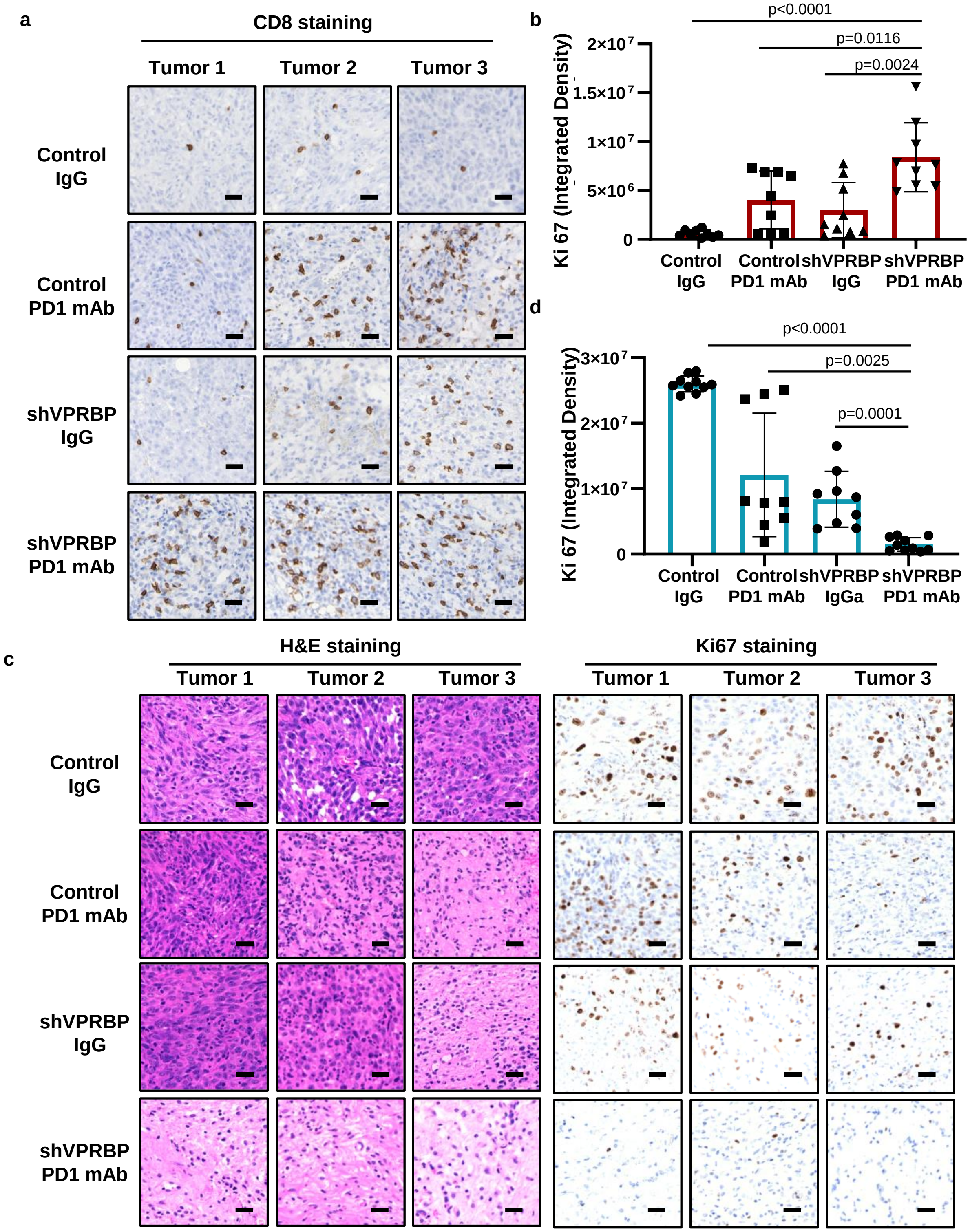

a
CD8 staining
Tumor 1
Tumor 2
Tumor 3
Control IgG
Control PD1 mAb
shVPRBP IgG
shVPRBP PD1 mAb
b
Ki 67 (Integrated Density)
p<0.0001
p=0.0116
p=0.0024
0
5×10^6
1×10^7
1.5×10^7
2×10^7
Control IgG
Control PD1 mAb
shVPRBP IgG
shVPRBP PD1 mAb
d
Ki 67 (Integrated Density)
p<0.0001
p=0.0025
p=0.0001
0
1×10^7
2×10^7
3×10^7
Control IgG
Control PD1 mAb
shVPRBP IgGa
shVPRBP PD1 mAb
c
H&E staining
Ki67 staining
Tumor 1
Tumor 2
Tumor 3
Control IgG
Control PD1 mAb
shVPRBP IgG
shVPRBP PD1 mAb

Supplementary Fig. 7. a, Representative immunohistochemistry images of CD8 staining in EMT6 control and shVPRBP tumors treated with IgG isotype or PD1 mAb. Scale bar: 25μM. b, Quantification of CD8 staining (n=3 tumors per cohort, 3 fields per section), mean±SD, two-way unpaired t test. P values were determined by unpaired two-tailed t test. c, Representative histological images of EMT6 tumors treated with IgG isotype, PD1 mAb, shVPRBP plus IgG isotype and shVPRBP plus PD1 mAb. . Scale bar: 25μM. d, Quantification of Ki67 staining (n=3 tumors per cohort, 3 fields per section), mean±SD, two-way unpaired t test. P values were determined by unpaired two-tailed t test. Source data are provided in the Source data file.

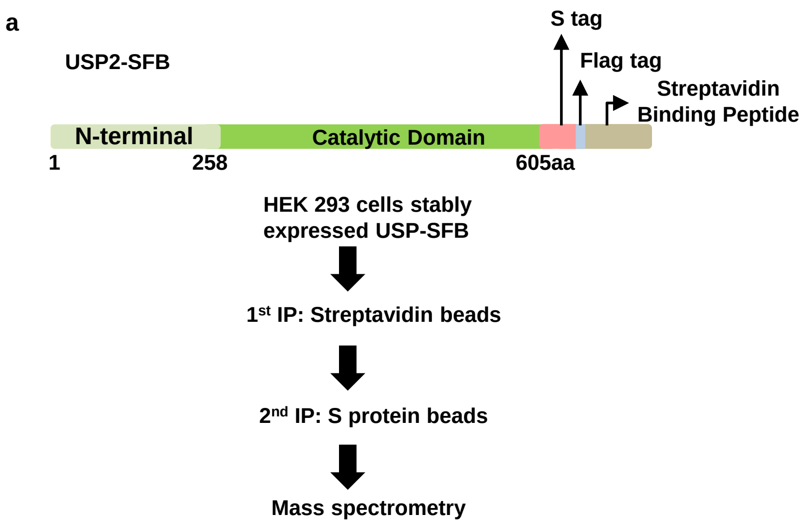

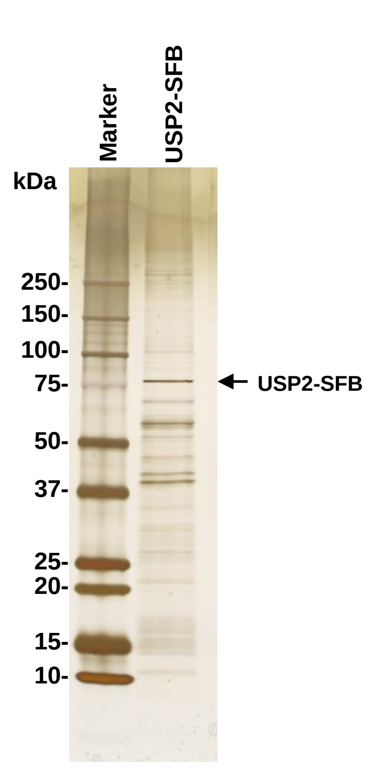

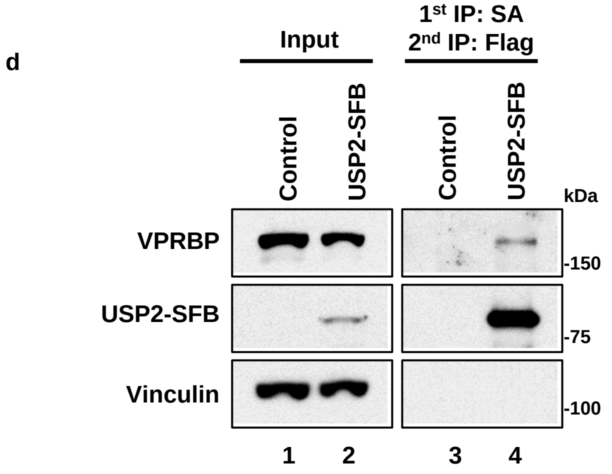

c

Identified VPRBP peptide by MS
K.GLGETATVLTK.E

Supplementary Fig. 8. a, Schematic illustration of USP2-SFB protein and the tandem affinity purification-mass spectrometry. b, Silver staining of the USP2-SFB complex. c, VPRBP peptide identified in USP2-SFB complex by Mass spectrometry(MS). d, Western blot analysis for VPRBP in HEK293 control and USP2-SFB cells subjected to tandem affinity purification as indicated. Data are representative of two independent experiments. Source data are provided in the Source data file.

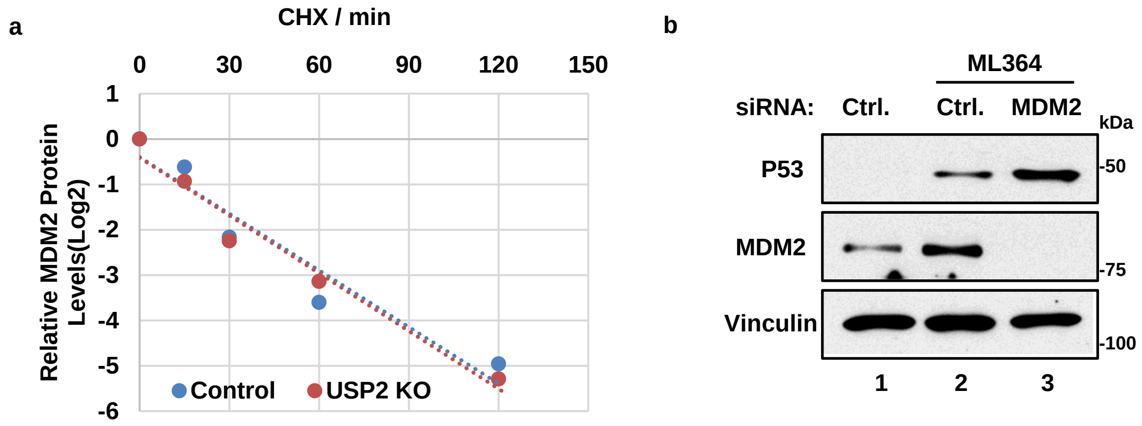

Supplementary Fig. 9. a, H1299 control and USP2 knockout (KO) cells were treated with 100μg/ml cycloheximide (CHX) for indicated time. Whole cell extracts were subjected to SDS-PAGE followed by western blot analysis. MDM2 protein abundance was quantified with ImageJ. b, Western blot analysis for p53 and MDM2 in U2OS cells that were transfected with control (Ctrl.) or MDM2 siRNA followed by 10μM of ML364 treatment for 24h. Data are representative of two independent experiments. Source data are provided in the Source data file.

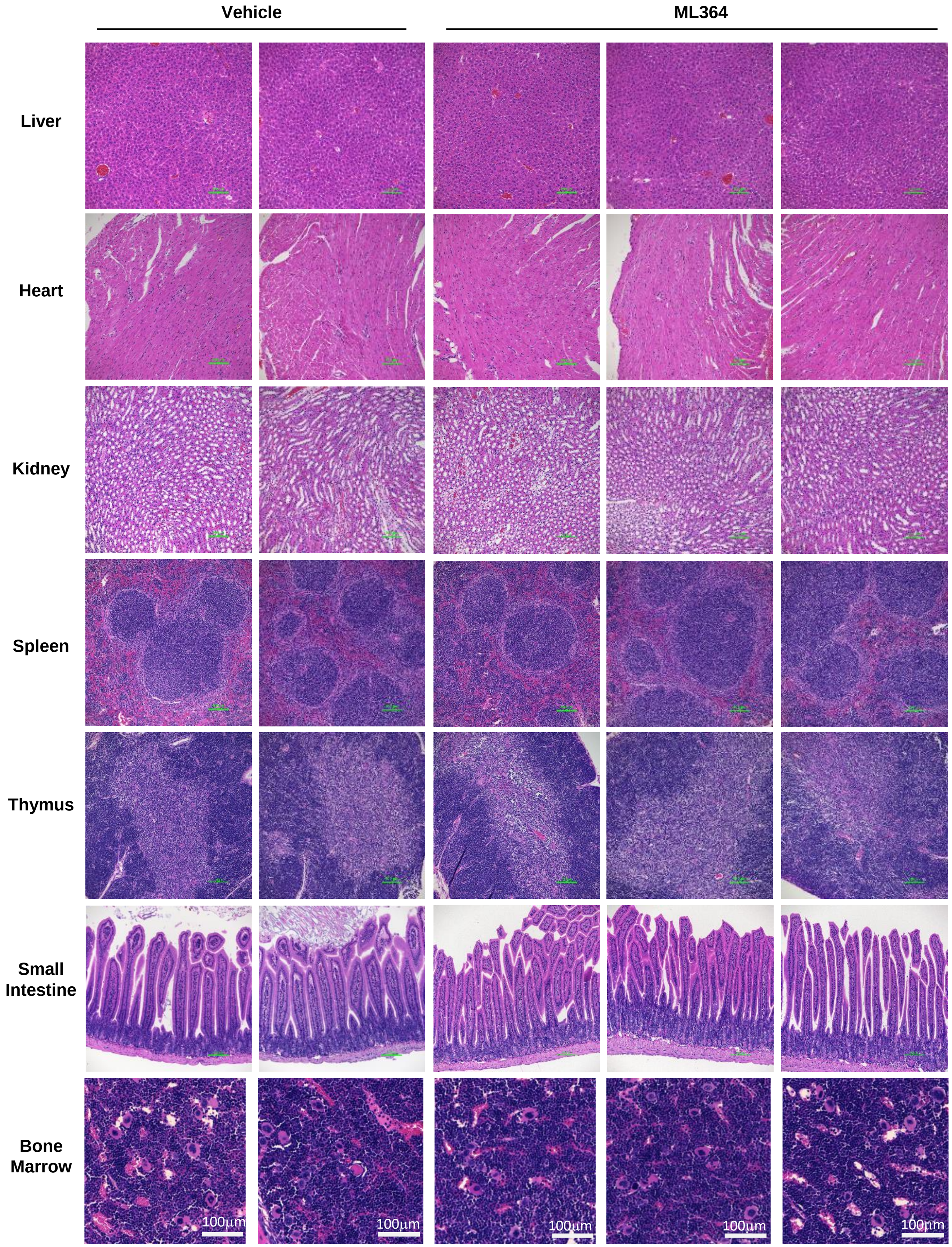

Supplementary Fig. 10. Representative images of Hematoxylin and Eosin (H&E) staining in indicated tissues of Balb/c mice treated with vehicle or 30mg/kg ML364 for 10 days. n=5 mice per cohort. Scale bar: 100μM . Source data are provided in the Source data file.

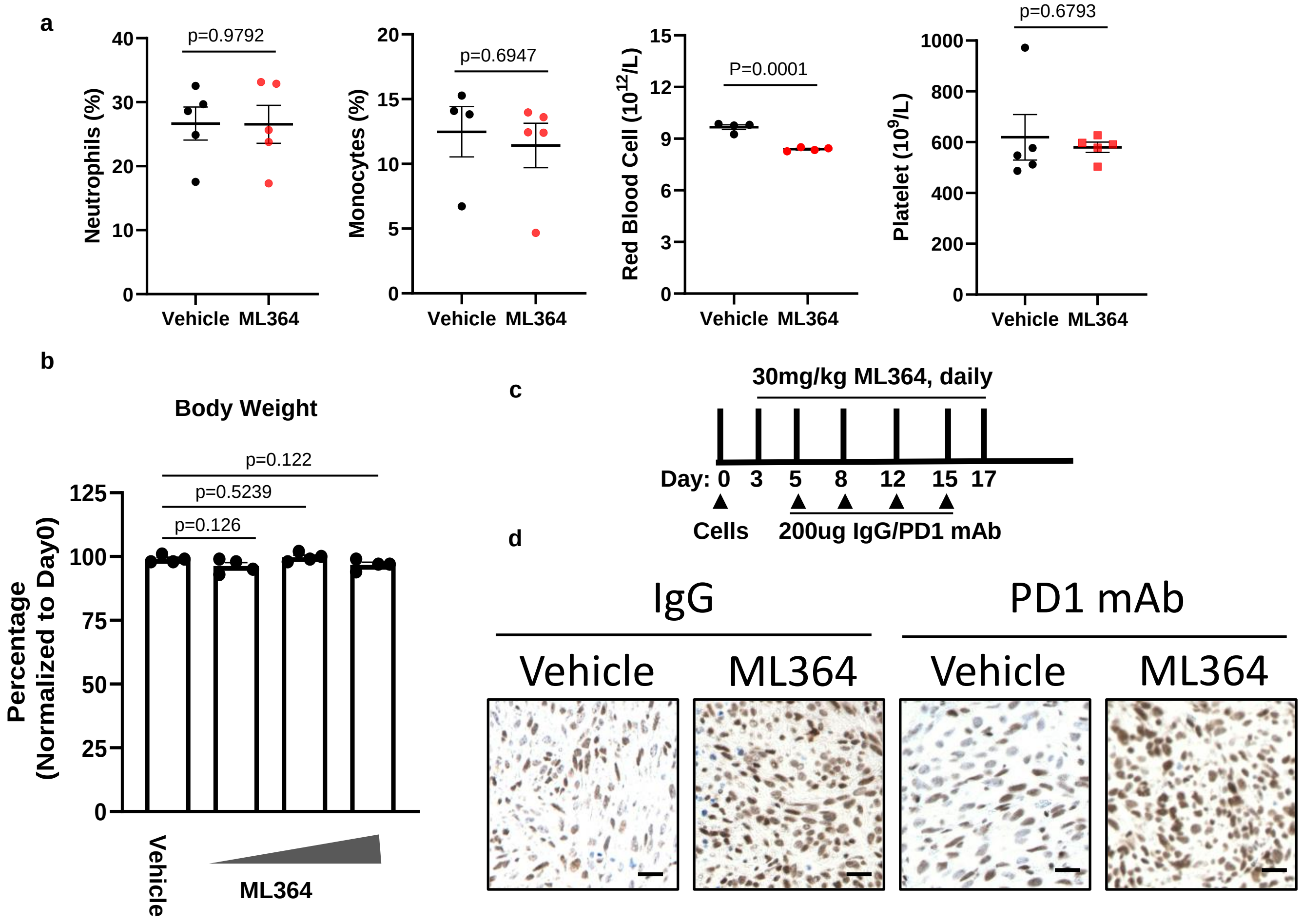

Supplementary Fig. 11. a, Complete blood count of Balb/c mice treated with vehicle or 30mg/kg ML364 for 10 days. n=5 mice, n(red blood cell) =4 mice, mean±SEM, two-tailed unpaired t-test. b, Weight change of Balb/c mice treated with vehicle, or 20, 30 and 40mg/kg ML364 for 4 days. n =4 mice, mean±SEM, two-tailed unpaired t-test. c, Schematic diagram of treatment plan in EMT6-impanted Balb/c mice. d, Representative immunohistochemistry images of PD-L1 staining in EMT6 tumors with indicated treatment. Scale bar: 25μM. n=3 images per group. Data are representative of two independent experiments. Source data are provided in the Source data file.

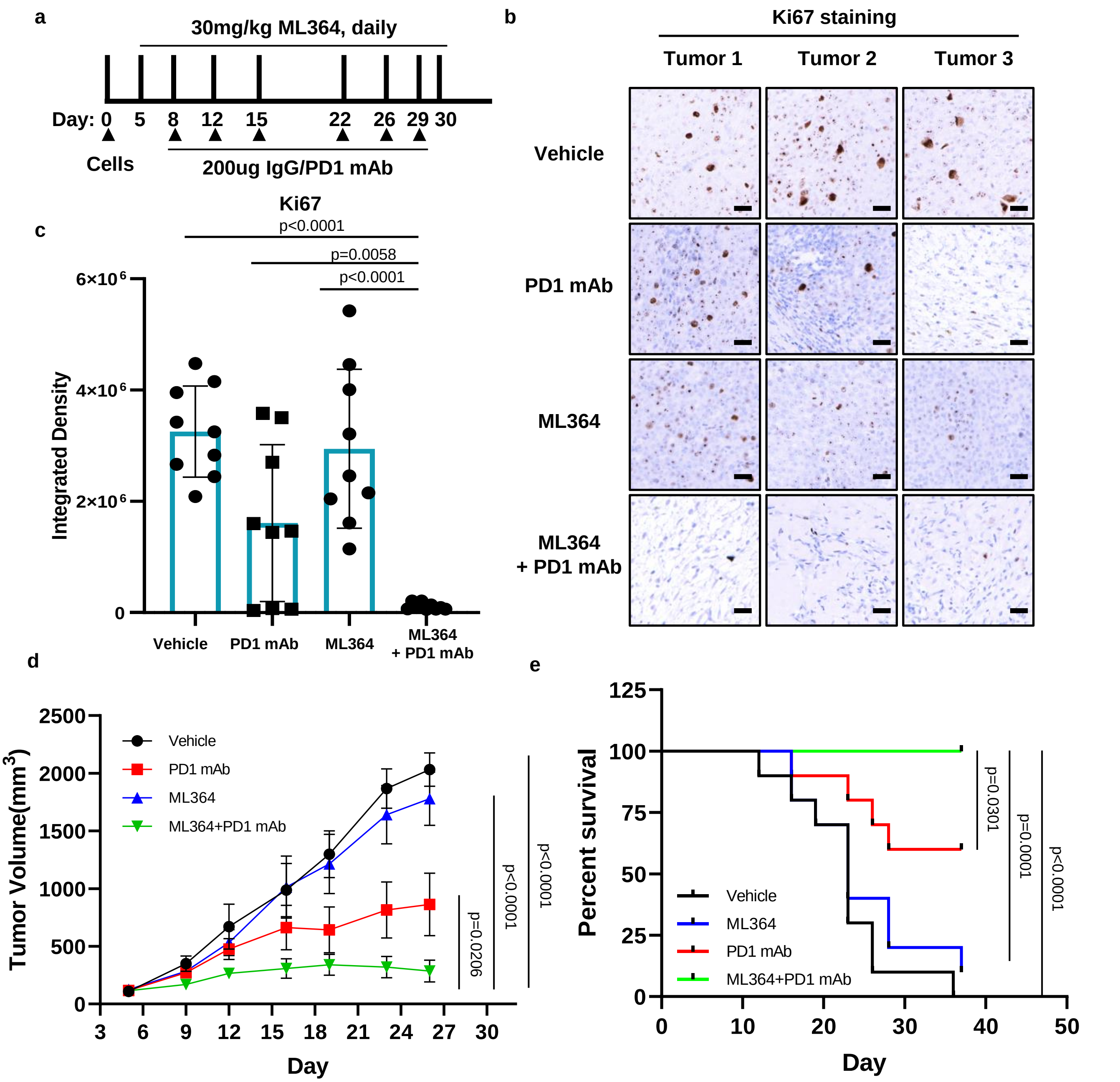

Supplementary Fig. 12. a, Treatment plan for RM-1-implanted C57BL/6 mice. b, Representative histological images of RM-1 tumors treated with IgG isotype, PD1 mAb, ML364 or ML364 and PD1 mAb. Scale bar: 25μM. P values were determined by unpaired two-tailed t test. c, Quantification of Ki67 staining (n=3 tumors per cohort, 3 fields per section) mean±SD, two-way unpaired t test. d, Mean tumor volume of RM-1-implanted C57BL/6 mice treated with indicated therapies. n=10, mean±SEM. P values were determined by two-way ANOVA. e, Kaplan Meier survival curve of RM-1-implanted mice treated with indicated therapy. n=10. P values were determined by log-rank test. . Source data are provided in the Source data file.

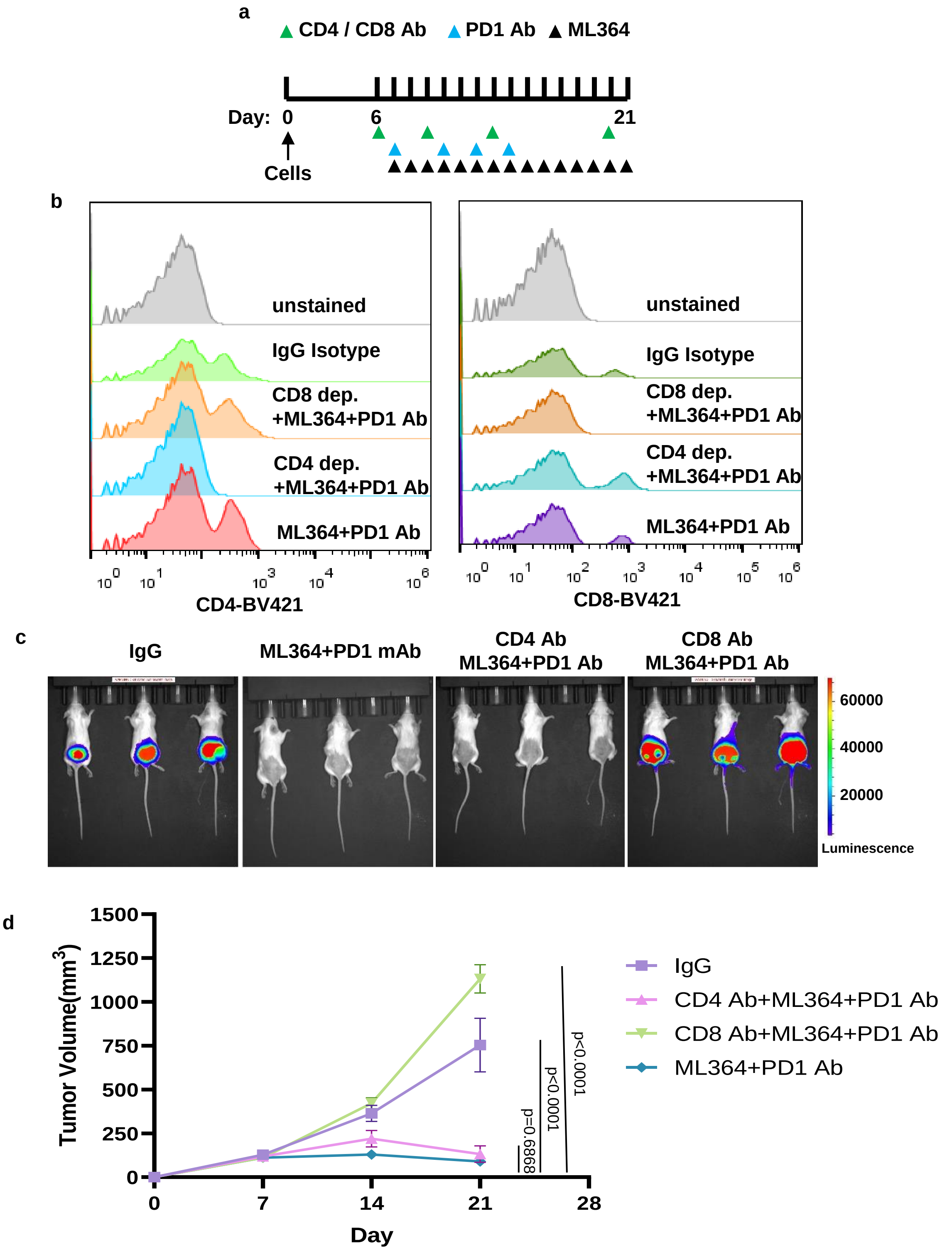

Supplementary Fig. 13. a, Treatment timeline for b-d. b, Mice were treated with antibodies as shown in a. FACS analysis of CD4+ (Left) and CD8+(Right) T cells in mice splenocytes. c, IVIS imaging of EMT6 tumors with the treatment as indicated. d, Tumor growth curve of EMT6 tumors with the treatment as indicated. n=10, mean±SEM, two-way ANOVA. P values were determined by two-way ANOVA . Source data are provided in the Source data file.

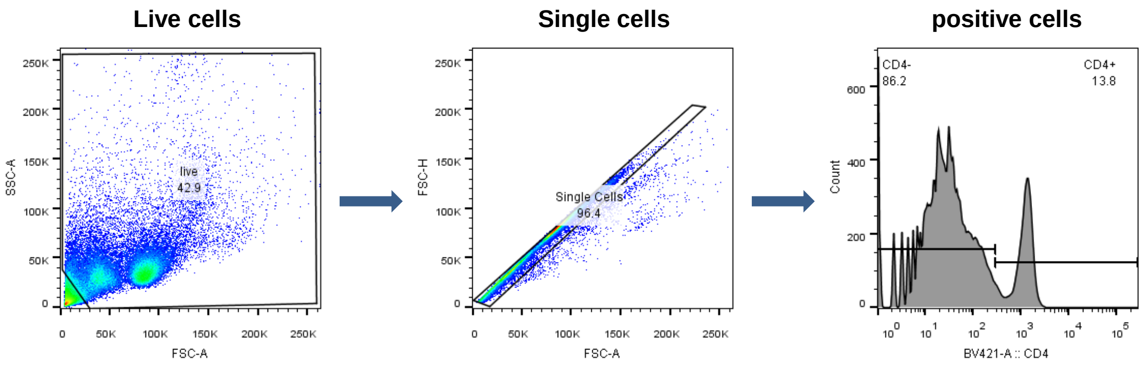

Supplementary Fig. 14. FACS gating strategy.
