# Supplementary material for: Targeting USP2 regulation of VPRBP-mediated degradation of p53 and PD-L1 for cancer therapy
Source: Nat Commun. 2023 Apr 6;14:1941. doi: 10.1038/s41467-023-37617-3 (PMC10079682; doi:10.1038/s41467-023-37617-3)
Supplement: Supplementary file 3 — Description of Additional Supplementary Files [file 41467_2023_37617_MOESM3_ESM.pdf]

Title: Supplementary Data 1

Description: Differentially expressed genes in USO2 and U2OS P53-null cells transfected with control or VPRBP siRNA.
