# Supplementary material for: Targeting USP2 regulation of VPRBP-mediated degradation of p53 and PD-L1 for cancer therapy
Source: Nat Commun. 2023 Apr 6;14:1941. doi: 10.1038/s41467-023-37617-3 (PMC10079682; doi:10.1038/s41467-023-37617-3)
Supplement: Supplementary file 4 — Reporting Summary [file 41467_2023_37617_MOESM4_ESM.pdf]

nature portfolio

Corresponding author(s): WEI GU

Last updated by author(s): Feb 27, 2023

Reporting Summary

Nature Portfolio wishes to improve the reproducibility of the work that we publish. This form provides structure for consistency and transparency in reporting. For further information on Nature Portfolio policies, see our Editorial Policies and the Editorial Policy Checklist.

Statistics

For all statistical analyses, confirm that the following items are present in the figure legend, table legend, main text, or Methods section.

n/a	Confirmed	
☐	☒	The exact sample size (*n*) for each experimental group/condition, given as a discrete number and unit of measurement
☐	☒	A statement on whether measurements were taken from distinct samples or whether the same sample was measured repeatedly
☐	☒	The statistical test(s) used AND whether they are one- or two-sided<br>*Only common tests should be described solely by name; describe more complex techniques in the Methods section.*
☒	☐	A description of all covariates tested
☐	☒	A description of any assumptions or corrections, such as tests of normality and adjustment for multiple comparisons
☐	☒	A full description of the statistical parameters including central tendency (e.g. means) or other basic estimates (e.g. regression coefficient) AND variation (e.g. standard deviation) or associated estimates of uncertainty (e.g. confidence intervals)
☐	☒	For null hypothesis testing, the test statistic (e.g. *F*, *t*, *r*) with confidence intervals, effect sizes, degrees of freedom and *P* value noted<br>*Give P values as exact values whenever suitable.*
☒	☐	For Bayesian analysis, information on the choice of priors and Markov chain Monte Carlo settings
☒	☐	For hierarchical and complex designs, identification of the appropriate level for tests and full reporting of outcomes
☒	☐	Estimates of effect sizes (e.g. Cohen's *d*, Pearson's *r*), indicating how they were calculated

Our web collection on statistics for biologists contains articles on many of the points above.

Software and code

Policy information about availability of computer code

Data collection	Applied Biosystems™ 7500 Fast Dx Real-Time PCR Instrument was used for QPCR; Attune™ NxT Flow Cytometer was used for FACS analysis; BD LSR II Flow Cytometer; IHC pictures were collected by Nikon ECLIPSE Ni Microscope; PerkinElmer IVIS Spectrum In Vivo Imaging System was used for bioluminescence.
Data analysis	Plots and statistical analysis was determined using Graphpad Prism8.2.1 or Microsoft Excel; Immunoblot densitometry was measured using ImageJ; FACS data were analyzed by Flowjo V10.

For manuscripts utilizing custom algorithms or software that are central to the research but not yet described in published literature, software must be made available to editors and reviewers. We strongly encourage code deposition in a community repository (e.g. GitHub). See the Nature Portfolio guidelines for submitting code & software for further information.

Data

Policy information about availability of data

All manuscripts must include a data availability statement. This statement should provide the following information, where applicable:

- Accession codes, unique identifiers, or web links for publicly available datasets
- A description of any restrictions on data availability
- For clinical datasets or third party data, please ensure that the statement adheres to our policy

The mass spectrometry data generated in this study have been deposited in the PRIDE (Proteomics IDEntifications Database) under accession code PXD040473 and PXD040477. The remaining data are available within the article and Supplementary Information. Source data are provided with this paper.

Human research participants

Policy information about studies involving human research participants and Sex and Gender in Research.

Reporting on sex and gender	n/a
Population characteristics	n/a
Recruitment	n/a
Ethics oversight	n/a

Note that full information on the approval of the study protocol must also be provided in the manuscript.

Field-specific reporting

Please select the one below that is the best fit for your research. If you are not sure, read the appropriate sections before making your selection.

☒ Life sciences ☐ Behavioural & social sciences ☐ Ecological, evolutionary & environmental sciences

For a reference copy of the document with all sections, see nature.com/documents/nr-reporting-summary-flat.pdf

Life sciences study design

All studies must disclose on these points even when the disclosure is negative.

Sample size	Sample sizes were determined to allow the statistical significance and based on the previous studies in the field (Zhang, J., Bu, X., Wang, H. et al. Nature 571, E10 2017; Mezzadra, R., Sun, C., Jae, L. et al.Nature 549, 106–110 2017; Lim S., Li C. et al. Cancer Cell 30, 925-939 2016.). Sample size of each experiment was provided in figure legends.
Data exclusions	No data was excluded from this study.
Replication	All experiments were repeated independently at least twice with similar results.
Randomization	Samples were divided into each group randomly in all experiments.
Blinding	Investigator was blinded to group allocation and data collection in in vivo experiments. Automated quantitative methods were used for IHC quantification to eliminate subjective interpretation of data. Blinding is not applicable for in vitro experiments in this study, because the same investigator performed cell culture, drug treatment and data analysis.

Reporting for specific materials, systems and methods

We require information from authors about some types of materials, experimental systems and methods used in many studies. Here, indicate whether each material, system or method listed is relevant to your study. If you are not sure if a list item applies to your research, read the appropriate section before selecting a response.

Materials & experimental systems

n/a	Involved in the study
☐	☒ Antibodies
☐	☒ Eukaryotic cell lines
☒	☐ Palaeontology and archaeology
☐	☒ Animals and other organisms
☒	☐ Clinical data
☒	☐ Dual use research of concern

Methods

n/a	Involved in the study
☒	☐ ChIP-seq
☐	☒ Flow cytometry
☒	☐ MRI-based neuroimaging

Antibodies

Antibodies used

Following primary antibodies were used for co-IP assay and western blot analysis: anti-USP2 (1:1000 dilution, Abgent Cat# AP2131c, RRID:AB_2212429); anti-PD-L1 (1:1000 dilution, Cell Signaling Technology Cat# 29122, RRID:AB_2798970); anti-PD-L1 (1:1000 dilution, Cell Signaling Technology Cat# 13684, RRID:AB_2687655); anti-VPRBP (1:2000 dilution, Bethyl Cat# A301-888A, RRID:AB_1524107); anti-VPRBP (1:1000 dilution, Santacruz Biotechnology Cat# sc-376850, RRID:AB_2905506); anti-mouse PD-L1 (1:1000 dilution, Abcam Cat# ab213480, RRID:AB_2773715); anti-IRF1 (1:1000 dilution, Cell Signaling Technology Cat# 8478, RRID:AB_10949108); anti-CUL4A (1:1000 dilution, Cell Signaling Technology Cat# 2699, RRID:AB_2086563); anti-CUL4B (1:1000 dilution, Sigma-Aldrich Cat# HPA011880, RRID:AB_1847340); anti-HA(1:5000 dilution, Roche Cat# 11867431001, RRID:AB_390919): anti-IRF1 (1:1000 dilution, Santa Cruz Biotechnology Cat# sc-74530, RRID:AB_2126826); anti-p53 (1:1000 dilution, Santa Cruz Biotechnology Cat# sc-126, RRID:AB_628082); anti-mouse p53(1:1000 dilution, Leica Biosystems Cat# NCL-L-p53-CM5p, RRID:AB_2895247); anti-PUMA (1:1000 dilution, Santa Cruz Biotechnology Cat# sc-28226, RRID:AB_2064827); anti-p21 1:250 dilution, (Santa Cruz Biotechnology Cat# sc-53870, RRID:AB_785026); anti-Actin (1:5000 dilution, Sigma-Aldrich Cat# A5441, RRID:AB_476744); anti-Flag (1:2000 dilution, Sigma-Aldrich Cat# F3165, RRID:AB_259529); anti-vinculin(1:5000 dilution, Sigma-Aldrich Cat# V9131, RRID:AB_477629).

Following second antibodies were used for western blot: Peroxidase AffiniPure Goat Anti-Mouse IgG (1:5000 dilution, Jackson Immunoresearch Cat# 115-035-146, RRID:AB_2307392) and Peroxidase AffiniPure Goat Anti-Rabbit IgG (1:5000 dilution, Jackson Immunoresearch Cat# 111-035-045, RRID:AB_2337938).

IgG isotype and monoclonal antibody used in mouse models are as following: IgG isotype (200ng/mouse, Bio X Cell Cat# BE0089, RRID: AB_1107769); anti-mPD-1 (200ng/mouse, Bio X Cell Cat# BE0273, RRID: AB_2687796); IgG isotype (200ng/mouse, Bio X Cell Cat# BE0090, RRID:AB_1107780); mCD4 (200ug/mouse, Bio X Cell Cat#0003, RRID:AB_1107642) and mCD8 (200ug/mouse, Bio X Cell Cat#0061, RRID:AB_1125541).

Antibodies used for FACS analysis are as following: PE-PD-L1 (1:20 dilution, BioLegend Cat# 124308, RRID: AB_2073556); BV421-CD8a (1:20 dilution, BioLegend Cat# 100737, RRID: AB_10897101); BV421-CD4 (1:20 dilution, BioLegend Cat# 100437, RRID: AB_10900241) and APC-Granzyme B (1:20 dilution, BioLegend Cat# 372204, RRID:AB_2687028).

Validation

All antibodies used in this study are commercially available and have been validated by manufacturer. Any validation statements are available on the manufacturer's website. The RRID# of each antidoies is also provided here.
anti-USP2 (1:1000 dilution, Abgent Cat# AP2131c, RRID:AB_2212429);
anti-PD-L1 (1:1000 dilution, Cell Signaling Technology Cat# 29122, RRID:AB_2798970);
anti-PD-L1 (1:1000 dilution, Cell Signaling Technology Cat# 13684, RRID:AB_2687655);
anti-VPRBP (1:2000 dilution, Bethyl Cat# A301-888A, RRID:AB_1524107);
anti-VPRBP (1:1000 dilution, Santacruz Biotechnology Cat# sc-376850, RRID:AB_2905506);
anti-mouse PD-L1 (1:1000 dilution, Abcam Cat# ab213480, RRID:AB_2773715);
anti-IRF1 (1:1000 dilution, Cell Signaling Technology Cat# 8478, RRID:AB_10949108);
anti-CUL4A (1:1000 dilution, Cell Signaling Technology Cat# 2699, RRID:AB_2086563);
anti-CUL4B (1:1000 dilution, Sigma-Aldrich Cat# HPA011880, RRID:AB_1847340);
anti-HA(1:5000 dilution, Roche Cat# 11867431001, RRID:AB_390919):
anti-IRF1 (1:1000 dilution, Santa Cruz Biotechnology Cat# sc-74530, RRID:AB_2126826);
anti-p53 (1:1000 dilution, Santa Cruz Biotechnology Cat# sc-126, RRID:AB_628082);
anti-mouse p53(1:1000 dilution, Leica Biosystems Cat# NCL-L-p53-CM5p, RRID:AB_2895247);
anti-PUMA (1:1000 dilution, Santa Cruz Biotechnology Cat# sc-28226, RRID:AB_2064827);
anti-p21 1:250 dilution, (Santa Cruz Biotechnology Cat# sc-53870, RRID:AB_785026);
anti-Actin (1:5000 dilution, Sigma-Aldrich Cat# A5441, RRID:AB_476744);
anti-Flag (1:2000 dilution, Sigma-Aldrich Cat# F3165, RRID:AB_259529);
anti-vinculin(1:5000 dilution, Sigma-Aldrich Cat# V9131, RRID:AB_477629).
Peroxidase AffiniPure Goat Anti-Mouse IgG (1:5000 dilution, Jackson Immunoresearch Cat# 115-035-146, RRID:AB_2307392);
Peroxidase AffiniPure Goat Anti-Rabbit IgG (1:5000 dilution, Jackson Immunoresearch Cat# 111-035-045, RRID:AB_2337938);
IgG isotype (200ng/mouse, Bio X Cell Cat# BE0089, RRID: AB_1107769);
anti-mPD-1 (200ng/mouse, Bio X Cell Cat# BE0273, RRID: AB_2687796);
IgG isotype (200ng/mouse, Bio X Cell Cat# BE0090, RRID:AB_1107780);
anti-mCD4 (200ug/mouse, Bio X Cell Cat#0003, RRID:AB_1107642);
anti- mCD8 (200ug/mouse, Bio X Cell Cat#0061, RRID:AB_1125541);
PE-PD-L1 (1:20 dilution, BioLegend Cat# 124308, RRID: AB_2073556);
BV421-CD8a (1:20 dilution, BioLegend Cat# 100737, RRID: AB_10897101);
BV421-CD4 (1:20 dilution, BioLegend Cat# 100437, RRID: AB_10900241);
APC-Granzyme B (1:20 dilution, BioLegend Cat# 372204, RRID:AB_2687028).

Eukaryotic cell lines

Policy information about cell lines and Sex and Gender in Research

Cell line source(s)	EMT6 ATCC Cat# CRL-2755<br>293T ATCC Cat# CRL-3216;<br>H1299 ATCC Cat# CRL-5803;<br>U2OS ATCC Cat# HTB-96;<br>CAL33 Creative Bioarray Cat# CSC-C0479;<br>HUCCT1 Creative Bioarray Cat# CSC-C9200W;<br>SKBR3 ATCC HTB-30;<br>A549 ATCC Cat# CCL-185;<br>A375 ATCC Cat# CRL-1619;<br>H460 ATCC Cat# HTB-177;<br>SKBR3 ATCC Cat# HTB-30;<br>MDA-MB-435 ATCC Cat# HTB-129;<br>MDA-MB-231 ATCC Cat# HTB-26;<br>RM-1 ATCC Cat# CRL-3310;
Authentication	All cell lines were not authenticated.
Mycoplasma contamination	The cell lines were tested negative for mycoplasma contamination
Commonly misidentified lines (See ICLAC register)	No cell line used in the study was found in the databases of commonly misidentified cell lines that are maintained by ICLAC.

Animals and other research organisms

Policy information about studies involving animals; ARRIVE guidelines recommended for reporting animal research, and Sex and Gender in Research

Laboratory animals	Nu/Nu mice(6-8 weeks, Charles River, 088); Balb/c mice(6-8 weeks, Jackson Laboratory, 000651); C57BL/6J mice (6-8 weeks, Jackson Laboratory, 000664); All mice were bred in a pathogen-free facility with a 12h light/dark cycle at 20 degree+/-3 and 40%-50% humidity.
Wild animals	No wild animals were used in this study.
Reporting on sex	female nu/nu mice and Balb/c mice were used for EMT6 mouse models. Male C57BL/6J mice were used for RM-1 mouse model.
Field-collected samples	No field-collected samples were used in this study.
Ethics oversight	This study is compliant with the relevant ethical regulations for animal experiments. All experimental protocols were approved by the Institutional Animal Care and Use Committee (IACUC)of Columbia University .

Note that full information on the approval of the study protocol must also be provided in the manuscript.

Flow Cytometry

Plots

Confirm that:

- [x] The axis labels state the marker and fluorochrome used (e.g. CD4-FITC).
- [x] The axis scales are clearly visible. Include numbers along axes only for bottom left plot of group (a 'group' is an analysis of identical markers).
- [x] All plots are contour plots with outliers or pseudocolor plots.
- [x] A numerical value for number of cells or percentage (with statistics) is provided.

Methodology

Sample preparation	Tissues were dissected from mice and digested with Collagase at 37C for 1h. Single cell suspension were prepared with 40uM cell strainer, then subjected to staining with specific antibodies and flow cytometry.
Instrument	Attune NxT; LSRII.
Software	Flowjo V10
Cell population abundance	at least 10000 cells were counted in each samples.

Gating strategy	live cells--single cells---positive staining cells. A figure examplifying the gating strategy is provided.

✘ Tick this box to confirm that a figure exemplifying the gating strategy is provided in the Supplementary Information.
